# Ketonemia variability through menstrual cycle in patients undergoing classic ketogenic diet

**DOI:** 10.3389/fnut.2023.1188055

**Published:** 2023-07-27

**Authors:** Ludovica Pasca, Cinzia Ferraris, Monica Guglielmetti, Costanza Varesio, Martina Totaro, Claudia Trentani, Claudia Marazzi, Ilaria Brambilla, Elena Ballante, Marisa Armeno, Gabriela Reyes Valenzuela, Roberto H. Caraballo, Pierangelo Veggiotti, Anna Tagliabue, Valentina De Giorgis

**Affiliations:** ^1^Department of Child Neurology and Psychiatry, IRCCS Mondino Foundation, Pavia, Italy; ^2^Department of Brain and Behavioral Sciences, University of Pavia, Pavia, Italy; ^3^Department of Public Health, Experimental and Forensic Medicine, Human Nutrition and Eating Disorder Research Center, University of Pavia, Pavia, Italy; ^4^Department of Public Health, Experimental and Forensic Medicine, Ketogenic Metabolic Therapy Laboratory, University of Pavia, Pavia, Italy; ^5^Department of Pediatrics, Pediatric Clinic, Foundation IRCCS Policlinico San Matteo, University of Pavia, Pavia, Italy; ^6^Department of Political and Social Sciences, University of Pavia, Pavia, Italy; ^7^BioData Science Center, IRCCS Mondino Foundation, Pavia, Italy; ^8^Department of Nutrition, Ketogenic Therapy Program Coordinator at Hospital Prof. Dr. Juan P. Garrahan, Buenos Aires, Argentina; ^9^Department of Neurology, Hospital Prof. Dr. Juan P. Garrahan, Buenos Aires, Argentina; ^10^Children Hospital Department Scienze biomediche e cliniche, University of Milan, Milan, Italy

**Keywords:** ketonemia variability, classic ketogenic diet, menstrual cycle, ketogenic dietary therapies efficacy, drug resistant epilepsy, epilepsy, GLUT1-DS

## Abstract

**Introduction:**

Ketogenic dietary therapies (KDT) are well-established, safe, non-pharmacologic treatments used for children and adults with drug-resistant epilepsy and other neurological disorders. Ketone bodies (KBs) levels are recognized as helpful to check compliance to the KDT and to attempt titration of the diet according to the individualized needs. KBs might undergo inter-individual and intra-individual variability and can be affected by several factors. Possible variations in glycemia and ketone bodies blood levels according to the menstrual cycle have not been systematically assessed yet, but this time window deserves special attention because of hormonal and metabolic related changes.

**Methods:**

This study aims at searching for subtle changes in KBs blood level during menstrual cycle in female patients undergoing a stable ketogenic diet, by analyzing 3-months daily measurement of ketone bodies blood levels and glucose blood levels throughout the menstrual cycle.

**Results:**

We report the preliminary results on six female patients affected by GLUT1DS or drug resistant epilepsy, undergoing a stable classic ketogenic diet. A significant increase in glucose blood levels during menstruation was found in the entire cohort. As far as the ketone bodies blood levels, an inversely proportional trend compared to glycemia was noted.

**Conclusion:**

Exploring whether ketonemia variations might occur according to the menstrual cycle is relevant to determine the feasibility of transient preventive diet adjustments to assure a continuative treatment efficacy and to enhance dietary behavior support.

**Clinical trial registration:**

clinicaltrials.gov, identifier NCT05234411.

## 1. Introduction

Ketogenic dietary therapies (KDTs) are well-established, safe, non-pharmacologic treatments used for children and adults with drug-resistant epilepsy and other metabolic disorders (1–3). There are currently four major KDTs: the classic ketogenic diet (CKD), the modified Atkins diet (MAD), the medium chain triglyceride diet (MCT), and the low glycemic index treatment (LGIT). There have been 4 randomized controlled trials to date (3 with class III evidence, and one with class II evidence) focusing on efficacy of KDTs compared to medications or a placebo arm, which have led to recognition of KDTs as valid and safe treatments (4). Diet quality plays a vital role in the achievement and maintenance of optimal ketosis, thus, an individualized approach, constant monitoring and the assurance of a prompt interface with keto-team are fundamental (2, 5–8).

According to international guidelines, for patients with drug resistant epilepsy KDT should be continued for at least 3 months to evaluate its efficacy, and, if well tolerated and effective, it can be continued for years and even lifelong (4). For GLUT1 deficiency syndrome (GLUT1-DS), a treatable metabolic encephalopathy characterized by complex movement disorders, drug-resistant epilepsy, and cognitive impairment, KDT is recognized as the gold standard treatment (9) and patients undergoing KDT are likely to achieve an optimal control of seizures (10).

Classic ketogenic diet is a high fat, adequate protein and low carbohydrate normo-caloric diet (1). The high-fat regimen provides about 87%–90% of daily energy intake from lipids, which are processed into free fatty acids in the liver, then oxidized in mitochondria, producing high levels of acetyl-Coenzyme A (acetyl-CoA), which cannot be oxidized in the Krebs cycle. The excess acetyl-CoA is converted to ketone bodies (KBs): acetoacetate and subsequently to acetone and beta-hydroxybutyrate (BHB) (11). The KBs can cross the blood–brain barrier and are transported by monocarboxylic acid transporters to brain interstitial space, the glia and the neurons. In these tissues, KBs act as substrates in the Krebs cycle and respiratory chain, contributing to brain energy metabolism. Clinical magnetic resonance spectroscopy in pediatric patients on the ketogenic diet demonstrated measurable beta-hydroxybutyrate, with a strong correlation to beta-hydroxybutyrate blood levels (11). Although the mechanism by which KDTs exerts its anticonvulsant effects is unclear, steady-state blood levels of BHB have been shown to correlate with the degree of seizure control (12, 13). The importance of maintaining stable KBs is relevant as they constitute an alternative fuel for cerebral metabolism instead of glucose in GLUT1-DS patients (2, 14). The maximum levels of blood ketones are obtainable with use of a 4:1 or 3:1 CKD and eventually addiction of MCTs. However, KBs blood levels undergo a significant inter-individual and intra-individual variability due to several factors beyond diet composition and ketogenic ratio, such as hydration, infection, steroidal therapy, and physical activities (15). The persistence of catamenial seizures has been reported in epileptic women on Modified Atkins Diet (16) and a feasibility trial aimed at stabilizing ketone levels by increasing MCT fat intake though menstrual cycle has been performed (16). This evidence suggests the need for personalized monitoring of individuals for optimization of their diet, especially at the beginning of the treatment but during whole follow-up. Indeed, international guidelines recommend that KBs should be checked at home by parents several times per week, preferably at different times of the day (4). There are currently no data on possible variations in glucose blood levels and ketone bodies blood levels according to different phases of the menstrual cycle in patients undergoing CKD. And, conversely, there is no data in literature about possible variations of the menstrual cycle induced by CKD. Whether a variability could actually occur should be worthy of investigation since reduced KBs levels could lead to increased seizure presentation in patients with drug resistant epilepsy and even movement disorder manifestation or increased fatigue and reduced attention in patients with GLUT1-DS. Thus, we believe it might be clinically relevant to assess possible patterns of variation of ketone bodies and glucose blood levels during menstrual cycle in patients undergoing KDT, considering a protective approach aimed at avoiding seizure exacerbation or overall clinical picture modifications whether fluctuations of ketone bodies and glucose blood levels in specific intervals of menstrual cycle will be demonstrated.

## 2. Materials and methods

This is a longitudinal multicenter study aimed at investigating the ketone bodies and glucose blood levels during menstrual cycle in female patients with a diagnosis of GLUT1-DS or drug resistant epilepsy undergoing CKD. Patients’ recruitment began in September 2021 and was performed at Mondino Foundation in Pavia, Ospedale dei Bambini V. Buzzi in Milan, and “Prof. Dr. Juan P. Garrahan” Hospital in Buenos Aires. The study protocol complied with the tenets of the Helsinki Declaration and was approved by the ethical committee of the IRCCS Policlinico San Matteo of Pavia on 12 June 2020 (code number: 20200047779). The protocol has been registered in clinicaltrials.gov (ID number NCT05234411 Name: KETOMENS, Ketonemia through the menstrual cycle).

### 2.1. Data collection

For each patient, baseline demographic and anthropometric data (age, height, weight, BMI circumferences, and body composition), clinical data (epilepsy etiology, epilepsy features and other neurological symptoms’ semiology and frequency, comorbidities, and general medical history), biochemical data (glycemia and BHB plasma level obtained from capillary blood and results of routine blood exams scheduled per follow-up), and therapeutic regimens (concomitant drug therapy, KD protocol, ketogenic ratio, compliance with diet prescription) were gathered. During the study, patients or caregivers were asked to compile a diary made up of 2 distinct sections: the clinical diary and the nutritional diary. The first one included information about the menstrual cycle (date of the menstrual period and its duration, possible symptoms, i.e., headache or stomach ache), ketone bodies and glucose blood levels, neurological symptoms (seizures, movement disorders, fatigue and/or a worsening of concentration skills) and physical activity (at rest/normal daily activities/physical activity). Ketone bodies and glucose blood levels were measured in fasting conditions (i.e., before meals) twice a day, both in the morning and in the evening, through a reflectometer, for 3 months. The nutritional diary serves to verify whether the patient is correctly following the dietary prescription and to rule out any possible bias influencing glucose and ketone bodies blood levels.

### 2.2. Participants

The study recruited patients diagnosed with drug-resistant epilepsy and GLUT1 deficiency syndrome undergoing a stable ketogenic dietary therapies from at least 3 months, who started KDT after the conventional metabolic screening (4). Participants were enrolled during their regular follow-up visits and were instructed to complete the clinical and nutritional diary at home. Eligible participants were female patients aged 13 years or older, who had been undergoing a Classic Ketogenic Diet (CKD) for at least 3 months and thus previously diagnosed with Drug-resistant epilepsy, according to ILAE definition (17) or GLUT1-DS. Included patients must have a regular menstrual cycle to correctly estimate the menstrual cycle phases during the study observation and to exclude potential causes of hormonal abnormal profiles. A detailed participant inclusion and exclusion criteria are listed in Table 1.

**Table 1:** Inclusion and exclusion criteria.

Inclusion criteria	Exclusion criteria
Patients with drug resistant epilepsy or GLUT1-DS undergoing KD from at least 3 months before inclusion to the study	Patients who experienced secondary amenorrhea
Patients who had regular menarche at least 3 months before inclusion to the study	Patients who have irregular menstrual cycle
Absence of recognized endocrinologic problems/ disease	Pregnant patients

### 2.3. Data analyses

The quantitative variables were described as mean ± standard deviation (or median and quartiles when appropriate), categorical variables were described as row count and percentage. Subject-wise comparisons between two measures (value in menstrual phase vs. basal value) were performed unpaired Wilcoxon rank tests. The global analysis was performed with mixed effects models to take into account the repeated measures for each subject. The day and the status (menstrual phase vs. basal value) was considered as covariates.

## 3. Results

Six patients were recruited among the three participating Centers. Three patients were affected by GLUT1-DS and the others had drug-resistant epilepsy. Age range was 13–18 years. All were in the normal weight range and maintained it for the duration of the study. All of them were following a stable CKD for a long time interval (time range 1–8 years) when included in the study. The compliance to the ketogenic dietary therapy was high in the entire cohort according to the food diaries provided. See Table 2 for demographics and clinical data.

**Table 2:** Participants clinical records.

	Age	Diagnosis	Duration of KDT at time of evaluation	KD ratio	Symptoms during menstrual cycle
Patient 1	15 years	GLUT1DS	6 years	2.3:1	Fatigue
Patient 2	18 years	GLUT1DS	8 years	3:1	–
Patient 3	14 years	GLUT1DS	5 years	3:1	Fatigue
Patient 4	14 years	Drug resistant epilepsy Lennox like	4 years	4:1	Increased seizure frequency
Patient 5	13 years	Drug resistant epilepsy	1 year	4:1	–
Patient 6	14 years	Drug resistant epilepsy Lennox like	2 years 8 month	3:1	–

Two out of six patients (33,3%), both with a diagnosis of GLUT1-DS, were found to have an increased fatigue during days of menstruation. No changes in seizures or movement disorder manifestations, when present, were found in patients with GLUT1-DS according to menstrual period. In 1 out of 3 patients with DRE, an increase in seizure frequency was found during menstruations.

**Figure 1:**
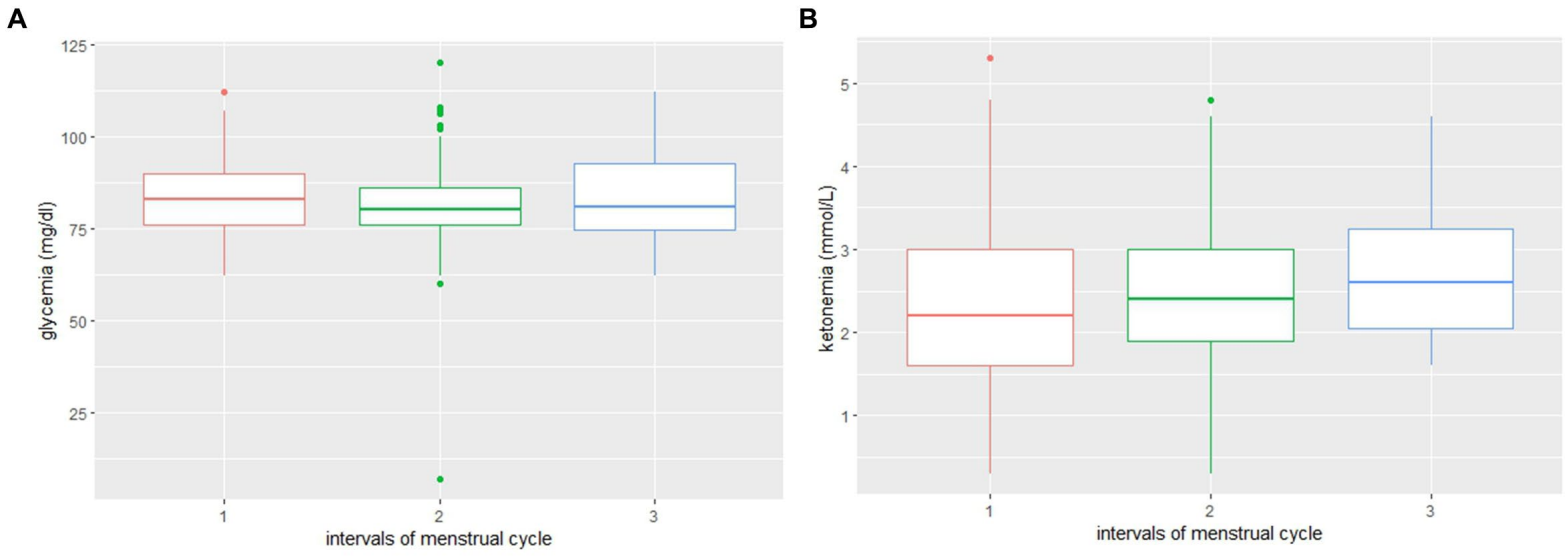
**(A)** Distribution of glycemia values during three phases: 1. Menstruations; 2. Non-menstruation days; and 3. Week before menstruations. **(B)** Distribution of ketone bodies blood level values during three phases: 1. Menstruations; 2. Non-menstruation days; and 3. Week before menstruations.

In the overall cohort, glycemia levels were found to be significantly higher (value of p 0.003) during menstruations compared to the remaining days. In the 7 days immediately before menstruations, the glycemia levels were found to be lower than in the remaining non menstrual days (value of *p* 0.0019) (see Figure 1).

Ketone bodies blood levels were found to be lower during menstruations in 4/6 patients, even if not statistically significant. See Table 3 for individual patients’ ketone bodies and glucose blood values.

**Table 3:** Individual ketone bodies and glucose blood level measurements.

	Glucose blood values during menstruations	Ketone bodies blood values during menstruations	Glucose blood values during non menstruations days	Ketone bodies blood values during non menstruations days
	Min-Max	Min-Max	Min-Max	Min-Max
	Mean	Mean	Mean	Mean
	Median	Median	Median	Median
	SD	SD	SD	SD
Patient 1	64–85 mg/dL	1.6–4.5 mmol/L	60–94 mg/dL	1.8–4.2 mmol/L
	76.07 mg/dL	2.7 mmol/L	74.77 mg/dL	2.94 mmol/L
	76 mg/dL	2.6 mmol/L	76 mg/dL	2.9 mmol/L
	5.96 mg/dL	0.72 mmol/L	6.91 mg/dL	0.54 mmol/L
Patient 2	62–107 mg/dL	0.3–2.2 mmol/L	67–98 mg/dL	0.3–1.9 mmol/L
	80.7 mg/dL	1.00 mmol/L	75.85 mg/dL	1.17 mmol/L
	80 mg/dL	1.00 mmol/L	76.5 mg/dL	1.30 mmol/L
	9.66 mg/dL	0.41 mmol/L	14.68 mg/dL	0.49 mmol/L
Patient 3	84–97 mg/dL	0.9–1.1 mmol/L	84–117 mg/dL	0.7–2.7 mmol/L
	90.6 mg/dL	1.13 mmol/L	93 mg/dL	1.5 mmol/L
	91 mg/dL	1 mmol/L	89 mg/dL	1.3 mmol/L
	5.3 mg/dL	0.4 mmol/L	7.2 mg/dL	0.5 mmol/L
Patient 4	75–112 mg/dL	1.6–5.3 mmol/L	62–120 mg/dL	1.3–4.6 mmol/L
	92.65 mg/dL	3.03 mmol/L	90.5 mg/dL	2.71 mmol/L
	92 mg/dL	3.05 mmol/L	91 mg/dL	2.70 mmol/L
	7.9 mg/dL	0.86 mmol/L	10.08 mg/dL	0.79 mmol/L
Patient 5	75–98 mg/dL	1.3–3.5 mmol/L	60–98 mg/dL	1.4–3.9 mmol/L
	86.41 mg/dL	2.31 mmol/L	82.55 mg/dL	2.45 mmol/L
	86.5 mg/dL	2.1 mmol/L	82 mg/dL	2.4 mmol/L
	6.65 mg/dL	0.63 mmol/L	6.96 mg/dL	0.56 mmol/L
Patient 6	70–83 mg/dL	1.5–4.7 mmol/L	62–80 mg/dL	1.4–4.8 mmol/L
	75.8 mg/dL	2.74 mmol/L	74 mg/dL	2.80 mmol/L
	75 mg/dL	2.1 mmol/L	74 mg/dL	2.5 mmol/L
	4.9 mg/dL	1.19 mmol/L	6.36 mg/dL	0.88 mmol/L

Physical activity, albeit mild, remained stable over time and therefore did not affect glycemia.

**Figure 2:**
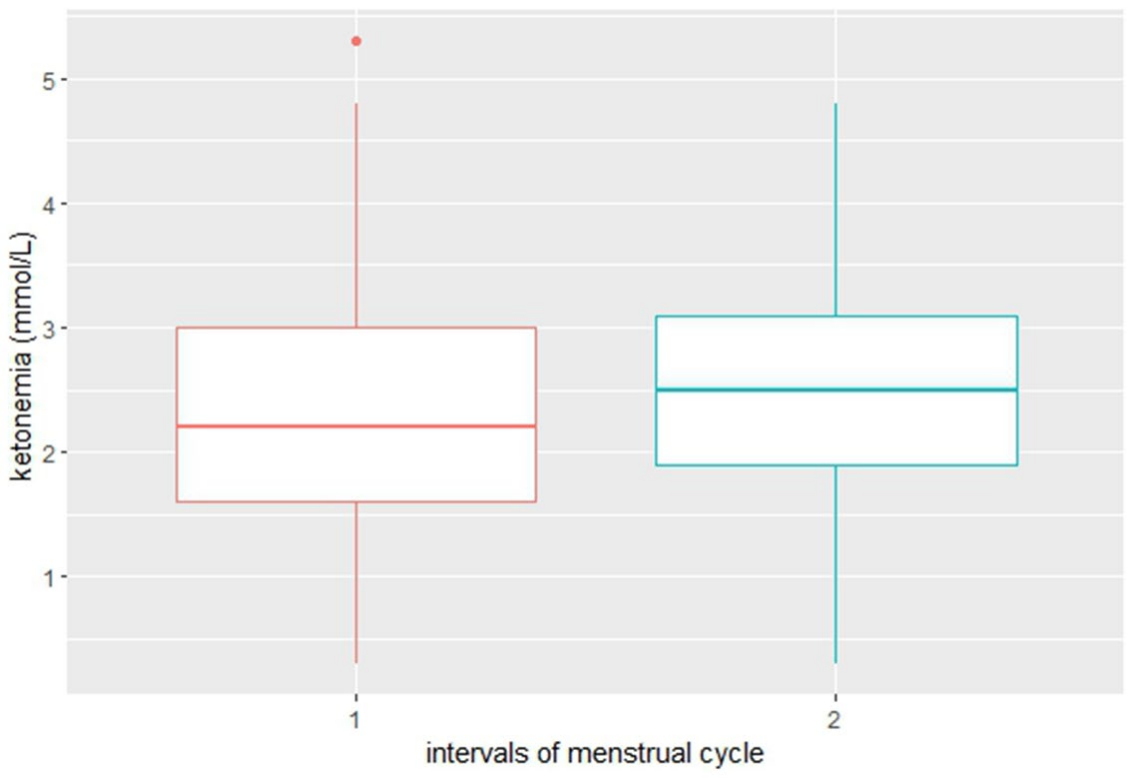
Distribution of ketone bodies blood level values during 1. Menstruations and 2. Non-menstruation days.

As far as ketone bodies blood levels, even if statistically significant variations were not observed, an inversely proportional trend compared to glycemia levels was documented (see Figure 2).

## 4. Discussion

To the best of our knowledge, the present study is the first one aimed at observing the course of KBs blood levels during the menstrual cycle in patients with GLUT1-DS and drug-resistant epilepsy undergoing KDT. Longitudinal studies serve in identifying changes in one or more variables between different periods, describing participants’ intra-individual and inter-individual changes over time and monitoring the degree and pattern of those changes (18). This is relevant for the proposed research since whether considered a reliable biomarker of KDT intervention, detecting whether KBs blood level changes are likely to occur in specific conditions, might lead to consideration of the suitability of transient preventive diet adjustments. Understanding this will also help to enhance dietary behavior support to assist patients in improving their diet quality.

Monitoring of urine and blood ketosis is recognized as helpful to check compliance to the KDT and to attempt titration of the diet according to the individualized needs. Nevertheless, it is not well understood how important ketosis is in achieving seizure and other possible disease symptoms control, since KBs may act via different mechanisms and blood KBs levels were not found to always correlate with seizure outcome (19).

There is a double rationale for investigating changes in ketone bodies and glucose blood level during the menstrual cycle. Firstly, during the luteal phase a reduction in glucose uptake related to the action of progesterone and increased insulin resistance have been documented (20–25). Secondly, interactions between seizures and menstrual cycle are possible, as suggested by variations in seizure frequency according to the day, phase and ovulatory status of the menstrual cycle, configuring “catamenial epilepsy” (26). The cyclic hormonal changes at the basis of catamenial seizure exacerbations are consistent with the neurophysiologic activity of estrogen and progesterone; indeed, for women with catamenial epilepsy who have regular menses, intermittent treatment approaches are thought to increase anti-seizure intervention during established phases of the menstrual cycle (27).

A prolonged CKD might have a reductive effect on glucose plasma level through a partial suppression of pancreatic action (28). A subject undergoing CKD, due to the chronic metabolic shift, in the presence of an increased energy requirement such as the one occurring during luteal phase, would utilize KBs as a preferential substrate, possibly decreasing their serum concentration. The increased requirement of fatty acids and cholesterol due to the yellow body could reduce KBs plasma level as well (20, 29). Other minor influencing factors could lead to KBs level fluctuations, such as a reduced food intake due to pre-menstruation discomfort or a minor compliance to the diet through the luteal phase, which implies increased carbohydrates consumption, especially in adolescents.

In the small cohort analyzed, significantly higher glycemic levels were found in the overall population during menstruation period. Even though not significantly lower in the menstruation period, ketone bodies blood levels were found to be lower during menstruations in the majority of patients analyzed. These findings support the hypothesis that blood ketone bodies levels and blood glucose levels might undergo inversely proportional subtle changes during the menstrual cycle. No evident clinical correlation was found with the biochemical data, except for seizure worsening during menstruation in one patient.

Whether patients with DRE undergoing KDT and likely to have catamenial exacerbations might benefit from an individualized treatment approach aimed at increasing ketogenic ratio in the time window of menstruation period remains to be investigated. The proposed study, for which preliminary results in a small cohort of six patients are presented, was designed to identify whether blood glucose and ketone bodies level variations are likely to undergo quantitative changes, considering that dietary therapies should be individualized. Broader population data are needed to assess variability rate and clinical implications.

This study has some limitations: first, the small size of the group of the participants was due to the difficulties encountered during subject recruitment. Besides the rarity of the pathologies considered, most of the patients would not have been able to collect the detailed data required, therefore they were not eligible for the study. Second, there is the absence of endocrinological and biochemical profiling (e.g., hormonal levels) but these observations might be scheduled in a different study, whether significant glucose and ketone bodies blood level variations according to menstrual cycle phases will be demonstrated. Third, the observation period is relatively short and data are auto-reported by the caregivers. Last, ketone bodies and glucose blood levels were measured through a reflectometer, which may be less precise in measuring these values but it is the mostly used tool for self-monitoring in clinical practice.

The present study has also some strengths: it is the first one in literature that evaluates the possible ketone bodies and glucose blood levels variations during the menstrual cycle in female patients undergoing KDTs. Second, it is a multicenter study, allowing to gather data from different countries and hospitals, despite the small sample size. Third, we included patients with a minimum CKD duration of 1 year, reducing the risk of ketone bodies fluctuations derived from other factors (i.e., the common variation that occurs in the first months of dietary therapy).

In conclusion, preliminary results showed a significant increase in glycemia levels during menstruation in the entire cohort and an inversely proportional trend of KB levels compared to glycemia. These data can be explained by several factors such as progesterone action and increased insulin resistance during menstruation, increase of energy requirement and thus KBs consumption during lethal phase, a reduction of KBs due to an increase of fatty acids utilization by yellow body. Importantly, not only a worsening of seizures might be a consequence of a reduction of ketone bodies blood level, but also other disease symptoms otherwise controlled by KDTs such as movement disorder, fatigue, concentration, and cognitive performance. Further research is needed to understand the role of ketosis in seizure and other disease symptoms control and thus the best ways to reach ketosis with an optimum balance of disease symptoms control and side effects.

## Data availability statement

The original contributions presented in the study are included in the article/supplementary files, further inquiries can be directed to the corresponding author.

## Ethics statement

The studies involving human participants were reviewed and approved by Ethical committee of the IRCCS Policlinico San Matteo of Pavia on 12 June 2020 (code number: 20200047779). Written informed consent to participate in this study was provided by the participants’ legal guardian/next of kin.

## Author contributions

CF and LP: conceptualization, methodology, and data curation. LP, CF, MG, MA, CT, CM, and CV: investigation. LP, CF, MG, CV, MT, and IB: writing—original draft preparation. LP, CF, MG, CV, MT, IB, EB, MA, GV, RC, PV, AT, and VDG: writing—review and editing. VDG and AT: supervision. VDG: project administration.

## Conflict of interest

The authors declare that the research was conducted in the absence of any commercial or financial relationships that could be construed as a potential conflict of interest.

## Publisher’s note

All claims expressed in this article are solely those of the authors and do not necessarily represent those of their affiliated organizations, or those of the publisher, the editors and the reviewers. Any product that may be evaluated in this article, or claim that may be made by its manufacturer, is not guaranteed or endorsed by the publisher.

## Abbreviations

KD: Ketogenic Diet
KDTs: Ketogenic Dietary Therapies
KBs: Ketone Bodies
GLUT1DS: GLUT1 Deficiency Syndrome
MCT: Medium Chain Triglycerides
MAD: Modified Atkins Diet
LGIT: Low Glycemic Index Treatment
MR: Magnetic Resonance
